# Knowledge and perceptions about non-communicable diseases by people living with HIV: a descriptive cross-sectional study from Chitungwiza Central Hospital Zimbabwe

**DOI:** 10.4314/ahs.v22i4.50

**Published:** 2022-12

**Authors:** Alexander Cheza, Boikhutso Tlou

**Affiliations:** 1 School of Nursing and Public Health, University of KwaZulu-Natal, College of Health Sciences, Durban, 4000, South Africa

**Keywords:** Non-Communicable Diseases (NCDs), HIV, Knowledge, Perceptions

## Abstract

**Background:**

Sub-Saharan Africa has shown a rise in morbidity and mortality due to NCDs. PLHIV have shown to be more exposed to NCDs and identifying the knowledge gaps might help the management of NCDs in PLHIV.

**Objective:**

The study was conducted in order to determine knowledge and perceptions regarding NCDs in PLHIV from Chitungwiza Hospital.

**Methods:**

This was a cross-sectional survey on 324 participants from Chitungwiza Hospital. Data collection was through a designed questionnaire. Knowledge and perceptions were evaluated, and the associated risk factors were identified using the Logistic Regression Model.

**Results:**

Results showed a 65% level of knowledge and 80% positive perceptions on NCDs. Participants <40 years of age were more knowledgeable (p=0.003) and history of NCD in the family influenced positively on knowledge (p=0.001). Females showed a more positive perception (p=0.043), both increasing age and low education negatively impacted the perceptions (p<0.001) as well as the knowledge (p=0.020).

**Conclusion:**

Knowledge and perception were moderately high, but reduced with decreasing levels of education and increasing age. The study recommends educational campaigns to disseminate information about NCDs in PLHIV, targeting the least educated population groups and those older than 40 years of age.

## Introduction

The Human Immunodeficiency Virus (HIV) is known for weakening the immune system, thus resulting in a higher exposure of patients to opportunistic infections, which led to a high mortality before the development of anti-retroviral therapy (ART); which significantly increased survival rates for people living with HIV (PLHIV)^1^. Non-communicable diseases (NCDs) have had a very high comorbidity in PLHIV thereby threatening to reverse the gains realised from ART^1^,^2^. The sub-Saharan African (SSA) region has been noted to be worst affected by HIV/AIDS, being home to almost two-thirds of the global PLHIV in 2017^3^,^4^. There has also been a notable increase in morbidity and mortality associated with NCDs globally, with LMICs equally affected. NCDs' mortality rate is more than 70% of the total deaths worldwide and more than two-thirds of these deathsoccur in LMICs^5^.

The World Health Organisation highlighted in 2016 that about 33% of all deaths in Zimbabwe were attributable to NCDs^6^. Thus, Zimbabwe is facing an increased risk of the comorbidity of NCDs and HIV disease burden^7^. The comorbidity rate is likely to be increased by an expanded lifespan after the introduction of ART, as well as the high urbanisation rate together with the associated risks involving lifestyle changes.

A literature scan reveals a dearth in studies focusing on knowledge, attitudes and perceptions on NCDs in LMICs and the SSA region. Most of the studies focusing on the knowledge, attitudes, and practices on NCDs were conducted in India^8^,^9^ and Pakistan^10^. To our knowledge studies conducted in the SSA region do not assess patients' knowledge and perceptions regarding the comorbidity of HIV and NCDs. For instance; a study in Uganda assessed the knowledge and attitudes of village health teams on NCDs,^11^ whilst a study in Cameroon focused on the knowledge, attitudes, and behaviour of the residents towards diabetes^12^. In Zimbabwe, a study by Chimberengwa and Naidoo focused on knowledge among rural residents and found a low level of knowledge about hypertension^13^. Therefore, knowledge about NCDs is not readily available to the public and hence affects their health seeking behaviour.

Despite the availability of global information about the growing burden of NCDs, strategies to address the escalation of NCDs in PLHIV remain evasive, especially in low-resourced countries^14^. An integrated approach which entails the involvement of all stakeholders, such as healthcare providers, the community, healthcare funders and the patients, amongst many others is necessary. PLHIV need knowledge to enable them to manage their exposure to NCDs by practising healthy lifestyles, as well as avoiding any risk factors which expose them to NCDs burden^15^. It is therefore apparent that there is limited evidence about how patients' knowledge and perceptions of HIV and NCDs may be incorporated into managing the mortality associated with the comorbidity or multi-morbidity of HIV and NCDs. The aim of the study is to assess the levels of knowledge and perceptions of PLHIV at Chitungwiza Central Hospital's (CCH) opportunistic infection clinic (OIC) regarding the comorbidity of HIV and NCDs to enhance management thereof. The study also takes a view of perceived challenges faced by patients in line with NCDs care. Thus, the study assesses the level of knowledge and perceptions with a view to inform strategies to be adopted to ensure PLHIV diagnosed with NCDs receive requisite care, management and/or treatment.

## Methodology

### Study Design

This is a cross-sectional explanatory study using a mixed methods approach to describe-the participants' responses. The study explores and descriptively documents the perceptions and knowledge of PLHIV on their exposure to the NCDs burden.

### Study Setting

The study was conducted at CCH (OIC), located in the town of Chitungwiza, which is in Zimbabwe's capital city metropolitan province of Harare, with an estimated population of about 1.5 million, serving circa 400 000 patients annually in both its in-patient and out-patient divisions. The CCH's catchment area spans 45 square kilometres in urban and peri-urban areas, and about 30 000 square kilometres in rural areas. The main sources of income for the patients served at CCH include self-help jobs, such as street vending for those in towns and market gardening for the peri-urban and rural populations. The CCH has been certified by the International Standards Organisation (ISO) since 2008.

### Study Population

The study population was made up of PLHIV receiving care from the CCH OIC. Therefore, the total number of such persons was 2500 people who received ART and other related care during the six-month data collection period. For participants' selection, the inclusion/exclusion criteria used.

### Sampling and Recruitment of Participants

Simple random sampling was used to select the patients who were receiving ART at the CCH OIC. Using the sample calculation tables by Krejcie and Morgan[16], a sample of 333 was determined from the population of 2500 patients seen by the CCH OIC during the six-month period of data collection. A significance level of 5% was used. The inclusion and exclusion criteria for the recruitment of the participants for the study are outlined below.

#### i Inclusion Criteria

- Patients who consented to the study;
- Patients aged 18 years or above as they were able to give their consent; and
- Only PLHIV who were on the CCH OIC register.

#### ii Exclusion Criteria

- All patients who were too sick and not able to communicate.

### Data Collection Procedures

Data was collected by trained resident nurses at the CCH OIC during the six-month period between August 2019 and January 2020. Consent was obtained from the respondents before their participation in the study. A questionnaire designed and distributed using the KoBo Toolbox was used for data collection. The questionnaire comprised of closed and open-ended questions. Participants provided their knowledge and perceptions regarding their conditions, as well as how they perceived the nature of the NCDs affecting them through the questionnaire. In a situation where direct and specific responses were required, closed-ended questions were used; in order to allow an infinite array of responses, open-ended questions were also used.

### Study Variables

#### Outcome variables

Knowledge and Perception – the study sought to ascertain knowledge and perceptions in PLHIV about their added exposure to NCDs and how NCDs affect them.

#### Explanatory variables

Demographic risk factors which according to literature include lifestyle factors, behavioural factors, religious factors, economic factors, and demographic factors, amongst many others^10^,^11^,^12^,^15^. The focus of this study was on demographic risk factors. Therefore, the explanatory variables for the study are gender, age and history of NCDs in the participants' families.

### Data Analysis

Data was analysed using a mixed methods approach. Stata Version 13 was used for data analysis and a p-value ≤ 0.05 was deemed statistically significant to the relationship between the dependent and independent variables. Descriptive quantitative results were presented using frequency distribution tables and bar graphs. Knowledge and perceptions of participants were assessed using different binary categorical questions. Frequency of responses for each question was used to compute the respective proportions. To determine the extent of how knowledgeable the participants were about NCDs, the authors adopted a subjective approach, as suggested by Ojo, Hawley and Desai^11^. The approach divides the calculated proportions into two categories (knowledgeable and not knowledgeable) using the following rule: ≥50% = knowledgeable and 0–49% = not knowledgeable. Two categories were also established for perception as follows: ≥50% = good perception and 0–49% = bad perception. Multiple logistic regression was used to determine the factors associated with participants' knowledge and perceptions.

Whilst for qualitative data, the study employed a six-step thematic analysis framework to analyse open-ended questions, which involved process of familiarising with the responses, generating themes, reviewing themes, defining themes, and writing up^17^. Major themes in terms of the challenges that impede the smooth care delivery for NCD conditions were identified under treatment adherence, financial and status of the home-based care. The focus of the open-ended questions was to establish the challenges faced by the participants in the care of HIV and NCDs and prompt further research into that. The results from the study are presented in the following section of this paper.

## Results

Data cleaning was performed prior to analysis and 9 incomplete questionnaires from respondents were removed from the 333 received, hence the responses analysed was from 324 participants. 60% of participants were female, whilst 40% were male. The highest represented participants were those older than 50 years (44%), whilst those aged between 18 and 25 years (4%) were the least represented. Emerging themes from the thematic analysis showed that under the theme ‘treatment adherence’ the participants had poor dose timetable adherence behaviours, another challenge was the lack of funds for medical care under the theme ‘financial’ and the third theme ‘status of home-based care’ showed that participants had unsupportive environments at home creating stigma.

Table 1 shows the NCDs that the participants were diagnosed with, either before or after commencement of ART. The results indicate that most of the 324 participants (52%) were diagnosed with hypertension, whilst 41% were diabetic. The least common NCD in participants was cancer diagnosed in 4% of participants. Results showed comorbidity of NCDs in 21% of PLHIV. However, the results did not specify the number of participants diagnosed with specific NCDs. Given the fact that some NCDs have genetics as a risk factor, the study also asked the question about the family history of the NCDs that they were diagnosed with. The results showed that 58.6% of participants had no other family member diagnosed with NCDs, whilst 41.4% had a history of similar NCDs in their families.

**Table 1:** Respondents' demographic and clinical characteristics

Features	Description	Frequency (Percentage)
**Age Group(years)**	18 – <25	13(4)
	25 - <40	87(27)
	40 – <50	81(25)
	≥ 50	143(44)
	**Total**	**324(100)**
**NCD Diagnosed**	Hypertension	168(52)
	Diabetes	133(41)
	Cardiovascular Diseases (CVDs)	78(24)
	Cancers	13(4)
	**Total**	**392(121)**
**Gender**	Male	129(40)
	Female	195(60)
	**Total**	**324(100)**
**Highest Education**	≤ Primary	61(19)
	Secondary	174(54)
	Tertiary	89(27)
	**Total**	**324(100)**
**History of NCDs in the Family**	Yes	134(41)
	No	190(59)
	**Total**	**324(100)**

**Figure 1:**
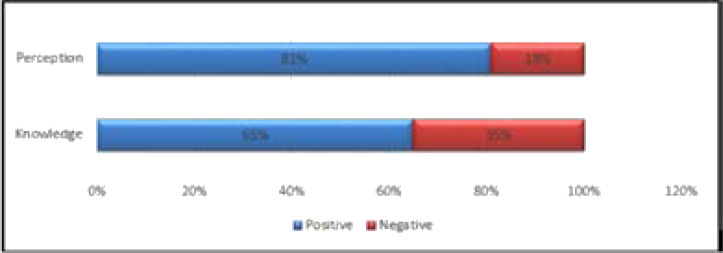
Overall Participants' Level of Knowledge and Perceptions on NCDs

Summary of the measurement of knowledge and perceptions are presented in Figure 1.

As shown in Figure 1, 35% of participants in the study were not knowledgeable about NCDs, whilst 65% were knowledgeable. Overall, the participants' perception of the comorbidity of NCDs in PLHIV is good (81% positive), with only 19% having negative perceptions about NCDs. The details of participants' perceptions are presented in Table 2 below.

**Table 2:** Participants' Perceptions and Knowledge on NCDs (n = 324)

Perceptions	Response	Frequency	Proportion	Rating
Can a non-communicable disease be spread in the community?	No	258/324	79.63	Good
	Yes	66/324	20.37	Bad
Can high blood pressure be silently fatal? Does it need to be checked regularly?	Yes	308/324	95.06	Good
	No	16/324	4.94	Bad
You must regularly go for medical examinations to screen for non-communicable diseases.	Yes	277/324	85.49	Good
	No	47/324	14.51	Bad
Heart disease is more common in active individuals than the elderly.	False	226/324	69.75	Good
	True	98/324	30.25	Bad
If NCDs are managed well, people can live normally and significantly improve their life expectancy.	True	300/324	92.59	Good
	False	24/324	7.41	Bad
In general, do you think NCDs are becoming common in Zimbabwe?	More Common	276/324	85.19	Good
	Less Common	48/324	14.81	Bad
**Knowledge**				
A stroke involves the cut of blood supply to the brain.	True	283/324	87.35	Good
	False	41/324	12.65	Bad
Diabetes mellitus can be inherited from parents.	True	288/324	88.89	Good
	False	36/324	11.11	Bad
Cancers affect only HIV-positive people.	False	251/324	77.47	Good
	True	73/324	22.53	Bad
Non-communicable disease cannot be managed.	False	213/324	65.74	Good
	True	111/324	34.26	Bad
You only suffer from one NCD at a time.	False	199/324	61.42	Good
	True	125/324	38.58	Bad

As shown in Table 2, the participants generally had good perceptions about NCDs. For instance, when asked if high blood pressure can be silently fatal and whether it needs to be checked regularly, more than 95% perceived that hypertension can be a silent killer and should therefore be tested regularly.

The study focused on assessing how knowledge and perceptions about NCDs are affected by demographic risk factors, using a binary logistic regression model. The independent variables (risk factors) under consideration in the study were gender, age, education, and history of NCDs in participants' families. The results of the model are given in Table 3.

**Table 3:** Demographic factors associated with knowledge and perceptions on NCDs

Variable	Categories	Knowledge				Perception			
		Odds Ratio	95% CI		P- value	Odds Ratio	95% CI		P- value
**Gender**	Female	1.662	0.116	3.220	0.079	1.833	1.006	2.659	0.043
	Male	1.000				1.000			
**Age**	≥40	0.606	0.273	0.934	0.003	0.108	0.047	0.158	<0.001
	<40	1.000				1.000			
**Highest Education**	≤ Primary	0.544	0.120	0.968	0.020	0.229	0.015	0.442	<0.001
	Secondary	0.963	0.434	1.492	0.059	0.991	0.402	1.580	0.066
	Tertiary	1.000				1.000			
**NCD** **Condition History in Family**	Yes	1.887	0.778	2.995	0.001	1.132	0.861	1.402	0.086
	No	1.000				1.000			

Table 3 summarises the demographic characteristics associated with the knowledge and perceptions of PLHIV receiving ART at the CCH OIC who participated in the study. The study findings showed that age, education and history of NCD condition in the family were significantly associated with knowledge of NCDs, whilst age, gender and education were significantly associated with perceptions about NCDs.

The odds of participants older than 40 years of age were 0.6 times less likely to be knowledgeable, as compared to those under 40 years (p-value = 0.003). Furthermore, the participants who attained a primary education level or less were 0.55 times less likely to be knowledgeable about NCDs when compared to those with a tertiary education (p-value = 0.020). Participants whose family members had a history of NCD conditions were 1.9 times more likely to be knowledgeable when compared to those with no history of NCDs in their families (p-value = 0.001).

On the other hand, female participants were 1.8 times more likely to have a good perception of the management of NCDs compared to male participants (p-value = 0.043). Furthermore, the odds of participants older than 40 years of age were approximately 0.1 times likely to have a good prception towards NCDs compared to those younger than 40 years (p<0.001). Participants who achieved a primary education level or less were about 0.2 times likely to have a good perception towards NCDs than those with a tertiary education (p<0.001). Gender and history of NCD conditions in the family were not significantly associated with the knowledge and perceptions thereof, respectively.

## Discussion

Data was collected from participants whose age range was wide although with the majority (44%) aged above 50 and most of the participants had a secondary level of education. The results showed that 65% of the participants were knowledgeable about NCDs and how they develop whilst 81% showed good perceptions about NCDs and their development. Findings showed that participants' moderately high knowledge was significantly influenced by age, education, and a history of NCDs in participants' families. On the other hand, perceptions were found to be significantly influenced by gender, education, and the age of participants. Results also showed a 21% comorbidity of NCDs in PLHIV.

The moderately high knowledge levels of PLHIV exhibited in the study are not consistent with other prior studies that have shown a significantly poor knowledge of NCDs^4^,^6^,^10^. The moderately high knowledge level exhibited by the participants is likely to have been a result of the demographics of the population served by the OIC, which is situated in a peri-urban setting. The level of knowledge shown by the participants was higher than other studies conducted in rural areas in Zimbabwe, such as research by Chimberengwa and Naidoo15 who conducted a study in a rural setting and found poor knowledge of hypertension, results that are inconsistent with this study findings. The level of knowledge was expected to be higher because of the peri-urban setting where the literacy level is higher. This was confirmed by the results, which showed that the level of knowledge is significantly influenced by age and education.

The moderately high knowledge levels about NCDs exhibited by the participants was however less than the level of good perception (81%). Findings revealed that the participants' perception about NCDs comorbidity are significantly influenced by age, gender, and education. Literature showed that most studies focused on knowledge, attitudes, and practices^11^–^15^. A few studies focusing on perceptions were conducted in developing countries^6^,^19^, which made it difficult for the researchers to compare this study's findings with other studies conducted in developing countries. However, these results were different from the results in studies conducted in developed countries such as Japan, where a study that focused on migrant workers' perceptions about NCDs found poor knowledge and perceptions due to language barriers^18^. This study's results were also different from the results obtained in a South African study, where the perception about NCDs and/or chronic diseases was found to be negative, both in urban and rural areas^15^,^17^. This study's results confirmed earlier findings that perception affects knowledge, but not all good perceptions translate into good knowledge^15^,^18^.

NCDs are preventable, especially through positive and health-conscious lifestyles. Therefore, given the relatively moderate knowledge gap of 35%, it is important to educate PLHIV and the general populace of Zimbabwe on the benefits of healthy lifestyles, especially in relation to NCDs^19^. This is important for closing the knowledge gap. PLHIV usually attend counselling sessions and support group meetings, which must be used to narrow the knowledge gap. The narrowing of the knowledge gap is expected to further narrow the perceptions gap regarding NCDs.

The participants highlighted challenges as applicable to their care in terms of HIV and NCDs; one issue pointed out was the adherence to medication timetables as the time slot for taking medication may arrive when participant is away from their medication or simply forgetting due to busy schedules. Literature shows a variance between people drawn from LMICs and high-income countries (HICs), with people from LMICs having poor medical adherence behaviour whilst those from HICs have higher adherence rates^4^, ^9^, ^11^. Several respondents pointed out that they are self-employed, and their normal day is usually never planned hence this may lead to challenges in remembering the times for medication. The other issue pointed out was not unique, financial crisis was also categorised as one of the thematic areas of concern. In as much some of these medications are dispensed free of charge at Government facilities there is still transport cost to get to those facilities as well as other indireccosts and all these expenses are amid the country's poor economic status which makes it costly to the patients. Furthermore, the participants pointed out stigma from relatives and community at large as another major challenge mainly due to limited knowledge of the conditions in the society. This shows need for community education on NCDs for the families and the public as highlighted in the key literature reviewed^6^, ^8^, ^9^, ^14^.

The strength of the study is that it focused on PLHIV, therefore critical in enhancing care delivery systems focused on minimising the mortality of NCDs in this specific cohort of PLHIV, who are at an increased risk to NCDs. One of the weaknesses is that the study is cross-sectional, and the results present a snapshot and do not show a long-term trend. In addition, the study was conducted at one hospital only, making the generalisation of findings difficult. The informative outcome of the study can benefit from making the study longitudinal, thereby assessing the long-term effects of knowledge and perceptions on NCDs as well as mitigating situational bias. Moreover, the study will benefit from expanding on the explanatory factors affecting the level of knowledge and perceptions on NCDs.

## Conclusion and recommendation

The study concludes that there is a moderately high knowledge (65 percent) about NCDs in the catchment area of the CCH OIC. However, the knowledge level exhibited was lower than perceptions of the participants about the NCDs in PLHIV, where the participants' perceptions were 81 percent. It is therefore imperative to further narrow the knowledge gap identified.

Given the findings obtained and the factors that significantly influence knowledge and perceptions, the authors recommend more education of PLHIV regarding NCDs. Enhancement of knowledge will help improve the lifestyles of PLHIV and help in reducing the mortality of NCDs in PLHIV. Training should embrace PLHIV with lower educational achievements as well as the elderly since these were found to negatively influence knowledge levels as compared to those with higher educational levels and the young generation respectively. Furthermore, healthy lifestyles are expected to minimise PLHIV's exposure to NCDs. Lessons on NCD risk factors should be incorporated into HIV counselling and support group sessions to enhance the knowledge levels of study participants.

## Data Availability

Data is available from the power-BI repository and can be accessed through the Principal Investigator.

## Abbreviations

ART: Anti-retroviral therapy
CCH: Chitungwiza Central Hospital
CVD: Cardiovascular disease
HIV: Human Immunodeficiency Virus
NCDs: Non-Communicable Diseases
OI: Opportunistic Infections
PLHIV: People Living with Human Immunodeficiency Virus
SSA: Sub-Saharan Africa
